# Comment on: Prognostic Effect of Liver Resection in Extended Cholecystectomy for T2 Gallbladder Cancer Revisited: A Retrospective Cohort Study with Propensity-Score-Matched Analysis

**DOI:** 10.1097/AS9.0000000000000344

**Published:** 2023-11-02

**Authors:** Wei Zhang, Yifan Wu, Yifan Yang, Zhong Chen, Tian Yang

**Affiliations:** From the * Department of Hepatobiliary Surgery, Affiliated Hospital of Nantong University, Nantong, Jiangsu, China; † Department of Hepatobiliary and Pancreatic Surgery, General Surgery Center, First Hospital of Jilin University, Changchun, Jilin, China.

We read with great interest the article by Park et al^1^ recently published in your esteemed journal. The authors have conducted a significant investigation into the impact of lymph node dissection with liver resection (LND+L) and lymph node dissection only (LND) on survival outcomes in T2 gallbladder cancer patients. They performed propensity-score matching (PSM) analysis to adjust for clinicopathological differences between the 2 groups, which is a commendable approach to reduce the effect of selection bias. We appreciate the authors’ meticulous work in this complex area of research. However, upon careful reading, our team has some concerns that we believe warrant further clarification.

First, the authors did not provide the number of T2 gallbladder cancer patients who underwent only cholecystectomy or cholecystectomy with liver resection but without LND in their 3 participating centers during the same period. This information is crucial for understanding the actual surgical practices for T2 gallbladder cancer in these centers.

Second, while the authors have stated that they conducted 2:1 PSM using factors including age, sex, operative method, T stage, lymph node metastasis, and adjuvant treatment, a detailed description of the PSM process and a comprehensive evaluation of the matching results were not provided. Surprisingly, despite the imbalance in many variables between the 2 groups, the LND+L group was able to maintain a sample size of 50 after 2:1 matching, with no loss of cases after matching. Based on our previous experience, this seems difficult to achieve unless the caliper for PSM is quite liberal. Unfortunately, the authors did not provide any description regarding this. As such, this leaves readers unable to assess the quality of the PSM, which is crucial for understanding the reliability of the study results.^2^

Third, in Table 1 of this study by Park et al^1^ (Supplemental Table 1 http://links.lww.com/AOSO/A264), more surprisingly, the *P* value for the operative method (open *vs.* laparoscopic/robotic) remained less than 0.001 after matching. This suggests that this PSM-related variable was not well balanced, contrary to the expectations in a successful PSM where covariates should be balanced across the treatment and control groups.^3^

Finally, we noticed that the sample size of 197 patients may not be sufficient to support the conclusions drawn. Furthermore, there is no explicit mention of how missing data were handled, which could introduce bias if not properly addressed.

We kindly suggest the authors provide a thorough clarification and explanation regarding these issues. This would greatly enhance the transparency and reproducibility of the study and allow readers to better interpret the findings.

## Acknowledgments

Study concepts: W.Z., Y.W., Y.Y., T.Y.; Preparation: W.Z., Y.W., Y.Y.; Editing: W.Z., Y.Y., Y.Y; Review: Z.C, T.A. All the authors reviewed the article and approved the final version.

## Supplementary Material


