# Supplementary material for: Comment on: Prognostic Effect of Liver Resection in Extended Cholecystectomy for T2 Gallbladder Cancer Revisited: A Retrospective Cohort Study with Propensity-Score-Matched Analysis
Source: Ann Surg Open. 2023 Nov 2;4(4):e344. doi: 10.1097/AS9.0000000000000344 (PMC10735063; doi:10.1097/AS9.0000000000000344)
Supplement: Supplementary file 1 [file as9-4-e344-s001.pdf]

Downloaded from http://journals.lww.com/annalsofsurgery by BhDMf5ePHKav1zEoum1tQfN4a+kJLhEZgbsIHo4XMi0hCywCX1AWnYQp/IlQrHD3i3D0OdRyi7TvSFl4Cf3VC1y0abggQZXdgGj2MwlZLeI= on 07/05/2023

Table 1. Baseline characteristics in LN dissection with liver resection (LND+L) and LN dissection only (LND) groups.

	Before Propensity-Score Matching				***After Propensity-Score Matching***			
	LND+L (*n* = 147)	LND (*n* = 50)	Total (*n* = 197)	*P*-value	LND+L (*n* = 100)	LND (*n* = 50)	Total (*n* = 150)	*P*-value
**Age (years)**	69 (61 - 75)	72 (63 - 77)	70 (62 - 76)	*0.033*	70 (61 - 76)	72 (63 - 77)	70 (63 - 77)	0.249
**Sex ratio (M:F)**	78:69	19:31	97:100	0.066	51:49	19:31	70:80	0.132
**BMI (kg/m^2)**	23.8 (21.5 – 25.8)	24.6 (21.8 – 26.4)	24.0 (21.5 – 25.9)	0.263	23.9 (21.6 – 26.0)	24.6 (21.8 – 26.4)	24.3 (21.6 – 26.0)	0.245
**ASA physical status grade**				0.065				0.081
I	29 (19.7)	3 (6.0)	32 (16.2)		20 (20.0)	3 (6.0)	23 (15.3)	
II	104 (70.7)	40 (80.0)	144 (73.1)		63 (63.0)	40 (80.0)	108 (72.0)	
III	14 (9.5)	7 (14.0)	21 (10.7)		12 (12.0)	7 (14.0)	19 (12.7)	
**CEA (ng/ml)**	1.7 (1.2 – 2.4)	2.1 (1.5 – 3.0)	1.8 (1.3 – 2.5)	0.499	1.7 (1.2 – 2.4)	2.1 (1.5 – 3.0)	1.8 (1.3 – 2.6)	0.073
**CA 19-9 (units/ml)**	12.0 (5.8 – 32.3)	12.0 (7.0 – 23.3)	12.0 (6.0 – 30.0)	0.223	12.0 (5.8 – 29.5)	12.0 (7.0 – 23.3)	12.0 (6.2 – 27.5)	0.634
**Operative method**				*<0.001*				*<0.001*
Open	109 (74.1)	4 (8.0)	113 (57.4)		62 (62.0)	4 (8.0)	66 (44.0)	
Laparoscopic/robotic	38 (25.9)	46 (92.0)	84 (42.6)		38 (38.0)	46 (92.0)	84 (56.0)	
**R status**				0.645				0.999
R0	143 (97.3)	48 (96.0	191 (97.0)		97 (97.0)	48 (96.0	145 (96.7	

ACCEPTED

Copyright © 2023 Wolters Kluwer Health, Inc. All rights reserved. Unauthorized reproduction of the article is prohibited.

		)				)	)	
R1	4 (2.7)	2 (4.0)	6 (3.0)		3 (3.0)	2 (4.0)	5 (3.3)	
**Tumor size (cm)**	4.0 (2.9 – 5.8)	4.0 (2.5 – 6.1)	4.0 (2.8 – 6.0)	0.863	4.1 (2.8 – 6.0)	4.0 (2.5 – 6.1)	4.0 (2.7 – 6.0)	0.869
**Tumor location**				0.463				0.635
Cystic duct/neck	18 (12.2)	3 (6.0)	21 (10.7)		10 (10.0)	3 (6.1)	13 (8.7)	
Body/fundus	109 (74.1)	40 (80.0)	149 (75.6)		73 (73.0)	39 (79.6)	112 (75.2)	
Both/entire	20 (13.6)	7 (14.0)	27 (13.7)		17 (17.0)	7 (14.3)	24 (16.1)	
**Histologic differentiation**				0.052				0.102
Well	47 (32.6)	13 (27.1)	60 (30.5)		37 (37.4)	13 (27.1)	50 (33.3)	
Moderate	70 (48.6)	28 (58.3)	98 (49.7)		46 (46.5)	28 (58.3)	74 (49.3)	
Poor	13 (9.0)	7 (14.6)	20 (10.2)		9 (9.1)	7 (14.6)	16 (10.7)	
**T stage**				*0.020*				0.064
T2a	66 (44.9)	32 (64.0)	98 (49.7)		48 (48.0)	32 (64.0)	80 (53.3)	
T2b	81 (55.1)	18 (36.0)	99 (50.3)		52 (52.0)	18 (36.0)	70 (46.7)	
**LN metastasis**	50 (34.0)	12 (24.0)	62 (31.5)	0.188	23 (23.0)	12 (24.0)	35 (23.3)	0.891
**Retrieved LNs**	8 (5 - 11)	7 (5 - 11)	8 (5 - 11)	0.689	8 (5 - 10)	7 (5 - 11)	8 (5 - 10)	0.914
**Lymphatic invasion**	64 (43.5)	23 (46.0)	87 (44.2)	0.903	40 (40.0)	23 (46.0)	63 (42.0)	0.733
**Venous invasion**	35 (23.8)	7 (14.0)	42 (21.3)	0.377	21 (21.0)	7 (14.0)	28 (18.7)	0.584
**Perineural invasion**	38 (25.9)	15 (30.0)	53 (26.9)	0.568	23 (23.0)	15 (30.0)	38 (25.3)	0.318

ACCEPTED

Downloaded from http://journals.lww.com/annalsofsurgery by BhDMf5ePHKav1zEoum1tQfN4a+kJLhEZgbsIHo4XMi0hCywCX1AWnYQp/IlQrHD3i3D0OdRyi7TvSFl4Cf3VC1y0abggQZXdgGj2MwlZLeI= on 07/05/2023

Copyright © 2023 Wolters Kluwer Health, Inc. All rights reserved. Unauthorized reproduction of the article is prohibited.

Downloaded from http://journals.lww.com/annalsofsurgery by BhDMf5ePHKav1zEoum1tQfN4a+kJLhEZgbsIHo4XMi0hCywCX1AWnYQp/IlQrHD3i3D0OdRyi7TvSFl4Cf3VC1y0abggQZXdgGj2MwlZLeI= on 07/05/2023

**Adjuvant treatment**								
Chemotherapy only	37 (25.2)	10 (20.0)	47 (23.9)	0.459	23 (23.0)	10 (20.0)	33 (22.0)	0.676
Radiotherapy only	2 (1.4)	0	2 (1.0)	0.999	2 (2.0)	0	2 (1.3)	0.553
CCRT	45 (30.6)	3 (6.0)	48 (24.4)	*<0.001*	16 (16.0)	3 (6.0)	19 (12.7)	0.083

BMI, body mass index; ASA, American Society of Anesthesiologists; CEA, carcinoembryonic antigen; CA 19-9, carbohydrate antigen; CCRT, concurrent chemoradiotherapy.
Values are presented as median (interquartile range) or n (%) unless otherwise indicated.

ACCEPTED

Copyright © 2023 Wolters Kluwer Health, Inc. All rights reserved. Unauthorized reproduction of the article is prohibited.
